# Development of a novel mesh model to define a new index “amount of stone” to evaluate calculus and predicting the lithotripsy time

**DOI:** 10.1007/s11255-023-03697-9

**Published:** 2023-07-13

**Authors:** Bingjian Wei, Yang Fu, Aidi Ma, Li Hong, Yunyan Wang, Shuo Gu, Lu Ji

**Affiliations:** 1 Department of Urology, Huai’an First Affiliated Hospital of Nanjing Medical University, No. 6 West Beijing Road, Huai’an, 223300 Jiangsu China; 2 Center of Lithotripsy, Huai’an First Hospital Affiliated of Nanjing Medical University, No. 6 West Beijing Road, Huai’an, 223300 Jiangsu China; 3 Department of Surgical Anesthesiology, Huai’an First Hospital Affiliated of Nanjing Medical University, No. 6 West Beijing Road, Huai’an, 223300 Jiangsu China

**Keywords:** Mesh model, Amount of stone, Percutaneous nephrolithotomy, Lithotripsy time, Volume of stone

## Abstract

**Objective:**

Develop a mesh model to define a new “index amount of stone” to evaluate calculus and predict lithotripsy time.

**Methods:**

The stones were divided into target units with diameter of 5 mm by the mesh from *x*, *y* and *z* directions, and the cross-sectional areas between units were calculated as amount of stone as a new index to evaluate calculus. Design a prospective study with 112 cases of percutaneous nephrolithotomy to verify the reliability of this index, and to compare the accuracy of the quantity, volume and maximum diameter of stones in predicting the time of lithotripsy.

**Results:**

Amount of stone (*Q*) is reliable. The lithotripsy time was significantly correlated with the amount of stone, volume and maximum diameter of the stone (*p* < 0.01). The three regression equations were valid. The linear fit in the amount group was larger than that in the volume group, and further larger than that in the maximum diameter group, with *R*^2^ values of 0.716, 0.661 and 0.471, respectively.

**Conclusions:**

It is more accurate and convenient to use amount of stone to evaluate calculus, which can be used to predict the lithotripsy time.

## Introduction

Urolithiasis incidence has kept rising worldwide [1]. More than 90% of urinary calculi can be treated by endoscopy. Large staghorn calculi can be cleared away by percutaneous nephrolithotomy [2, 3]. However, it remains unsolved about how to evaluate the stone size quickly and accurately before operation to reduce intraoperative complications, such as infection and bleeding, and guide surgical procedures.

No standards for preoperative evaluation of stone size have been established. Common indicators include maximum diameter, maximum cross-sectional area, volume and surface area. Among them, volume, which can be calculated by 3D technology, has been attached with high clinical value [4]. However, the complexity in calculation limits its wide use in clinic. Maximum diameter is also being used, but unscientific. In real-time surgery, clinicians are mainly concerned with the difficulty in breaking a stone to pieces of favorable sizes, rather than the simple maximum diameter, volume and other indicators.

Therefore, the author tries to establish a mesh model and use it to define a new index to evaluate stones.

Here are two questions to be resolved before the study.

1. What is the target size? There are often two modes of intraluminal lithotripsy: fragmented lithotripsy and powdered lithotripsy [5]. Each has its own advantages and disadvantages [6, 7]. For larger stones, fragmented lithotripsy can quickly crush stones, shorten operation time and reduce surgical complications [8]. Many scholars believe that a diameter of 4 mm is the target size of crushed stones, and those with a diameter below 4 mm are called meaningless residues [2]. Only stones with a diameter of less than 2 mm can become free [9]. We believe that the target size should be determined according to the surgical method. For example, in percutaneous nephroscopic surgery, the stones in the 24f standard channel only need to be broken into less than 7 mm in diameter before being washed out through the channel, so it is meaningless to break them into smaller sizes. Similarly, the stones in the 18f microchannel only need to be broken into pieces less than 5 mm in diameter. For operations in the ultramicro-channels or soft mirrors, the stones should be crushed into less than 2 mm in diameter.
2. How to define the cost of stone-crushing? Previous indicators cannot. For example, volume is used to describe the size of stones. If the target size is 4 mm in diameter, but there is a volume of stones 4 mm in diameter, and the cost of breaking them is 0, how do you define it? The volume of a regular-shape stone with a diameter of 8 mm is about 8 times that of 4 mm. Is the cost of crushing the former 8 times that of crushing the later? How about multiple stones? It is obvious that more scientific indicators are needed to solve these problems.

## Building the mesh model and defining the amount of stones

Fracture theory is the most widely used in establishing a stone-crushing model. Using this theory, we regarded stone fragmentation as a process making enough cracks in the stone to fragment it into pieces with the target size. We established a mathematical model to calculate the minimum number of cracks for crushing.

Considering that non-contrast CT, usually at a spacing of 5 mm, is commonly used for preoperative stone examination, we defined the target size as 5 mm in diameter, and a cubic-shaped stone with edges of 5 mm (on *x*, *y*, *z* axes, respectively) as a target stone unit. A stone was divided into multiple target stone units with a 5 mm mesh in three directions. The bottom surface of each target stone unit was defined as the target cross-sectional area (5 mm × 5 mm = 0.25 mm^2^, expressed in u), then the number of target cross-sections between two units was calculated, and the total number of cross-sections (*Q*) of each stone was obtained as the theoretical minimum of cross-sections.

**Fig. 1:**
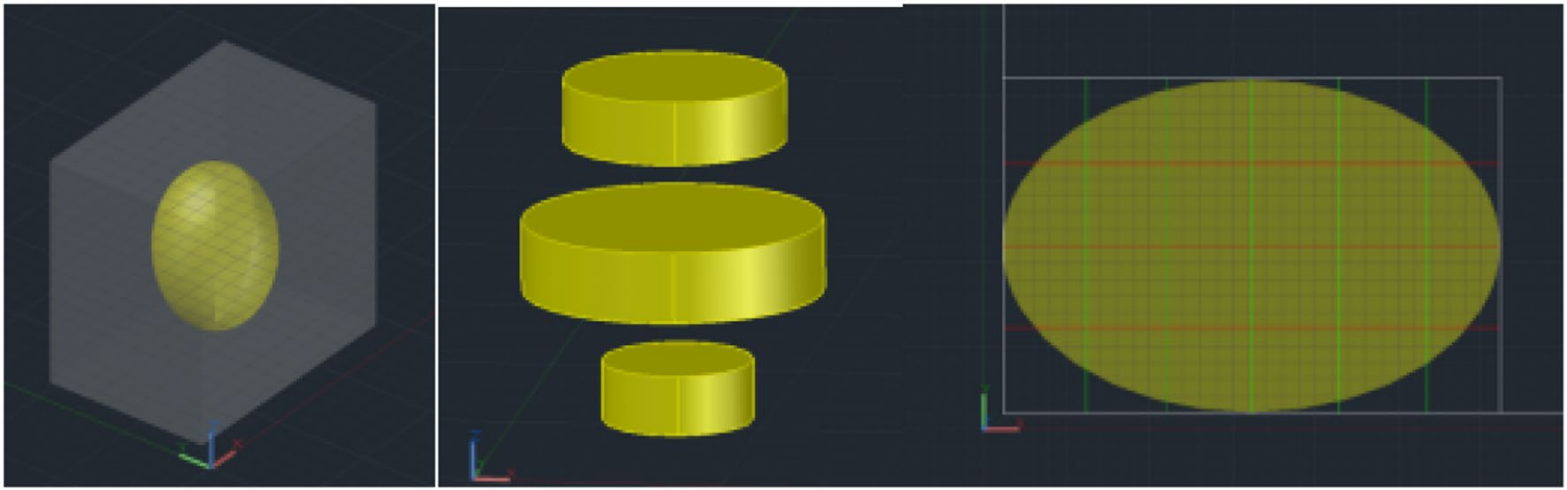
3D Schematic diagram of mesh model

Each level is measured with the orthogonal method. The *x* and *y* values at each level of CT are measured. *x* is the maximum diameter and *y* is the maximum diameter perpendicular to the *x*-axis. The *x* and *y* values are graded by 5 mm: 0 mm < *x* < 5 mm is expressed as 1; 5 mm ≤ *x* < 10 mm as 2; 10 mm ≤ *x* < 15 mm as 3. The same method is used for *y*. The measurement results are expressed by (*x*_1_, *y*_1_), (*x*_2_, *y*_2_), (*x*_3_, *y*_3_), and so on. Then, the number of cracks perpendicular to the *z*-axis (*n*_1-2_) is expressed as the product of *x* and *y*. The number of cracks parallel to the *z*-axis (*n*_1_) = *x*_1_(*y*_1_ − 1) + *y*_1_(*x*_1_ − 1), *n*_2_ = *x*_*2*_(*y*_2_ − 1) + *y*_2_(*x*_2_ − 1), *n*_3_ = *x*_3_(*y*_3_ − 1) + *y*_3_(*x*_3_ − 1), and so on. The total number of cross-sections of the stone is called the amount of the stone: *Q* (*u*) = *n*_1_ + *n*_1-2_ + *n*_2_ + *n*_2-3_ + *n*_3_ + …. (Fig. 1). If there are multiple stones, the *Q* of each stone is calculated and summed up. The *Q* is the theoretical minimum cost.

## Model validation

Next, we verified the reliability of this index from three points.

1. The mathematical basis of the model

The foundation of the model is that the stones are supposed to be regular. Because most of the stones are irregular, errors are inevitable. Some scholars believe that the elliptical formula can be used to calculate the cross-sectional area of stones [10], while others believe that the elliptical formula is inaccurate [11]. Therefore, we summed the cross-section areas of the stone measured layer-by-layer orthogonally, multiplied by the spacing, and compared the results with the CT 3D reconstruction volume, to verify whether the influence of the stone shape on the model was within the acceptable range.

2. The error of the *Q*

The selection of non-contrast CT sections has a bias which may lead to deviation in the calculation. Therefore, we measured two different CT planes to obtain two groups of results, and compared them to verify the error in the *Q*.

3. The superiority of *Q*

The *Q* is actually a sum of sectional areas, which can reflect the total laser energy used during the operation. This total energy is the energy of one single laser multiplied by laser times. However, clinicians do not care much about this total energy. They pay more attention to lithotripsy time. Therefore, we aimed to find the correlation between the amount of stones and the lithotripsy time, and analyze the superiority of amount of stone over volume and maximum diameter in predicting the lithotripsy time.

## Design of clinical study

We designed a prospective study to resolve the above three points. Since the stones with a diameter less than 5 mm can only be washed out directly through a channel of about 18F under the percutaneous nephroscope, we used percutaneous nephrolithotomy to verify the effectiveness of our new method. During the operation, the channels with a diameter of 18F and holmium laser were selected. Holmium laser is a solid-state pulsed laser using rare element as the excitation medium. The mechanism is that after the activation of the Holmium laser, the stone and its surrounding water absorbs the laser energy, causing the temperature to rise and the stone to undergo a thermochemical reaction. At the same time, the water around the stone generates plasma bubbles (or steam bubbles), and uses the shock wave generated in the expansion or crushing process to smash the stone [12].

## Materials and methods

A total of 198 patients who had received PCNL in our hospital from March 2021 to December 2022 were recruited and 112 eligible were included.

## Indicators and selection methods

General indicators included age, gender, height, weight, and BMI; amount of stone (*Q*), volume, cross-sectional surface area, and Hounsfield unit (HU) values determined in the non-contrast CT; postoperative calculus removal. Calculus removal was assessed by re-examining the plain images of the kidney–ureter–bladder (KUB) within 1 month after surgery. If no high-density shadows in the urinary tract on the surgery site or spot shadows with a diameter less than 5 mm were seen in the KUB, calculus removal was determined and the patient could be included. If high-density shadows with a diameter of 5 mm or more in the urinary tract on the surgery site were observed in KUB, calculus residue was considered and the patient should not be included.

## Clinical data

112 patients included 73 men (65.1%) and 39 women (34.9%). Their age was between 23 and 68 years, with a mean of 49.92 ± 11.36 years. The height was between 150 and 186 cm, with a mean of 166.56 ± 8.04 cm. The weight was between 47 and 105 kg, with a mean of 71.06 ± 12.43 kg. The BMI was between 18.73 kg/m^2^ and 32.05 kg/m^2^, with a mean of 25.51 ± 3.37 kg/m^2^. The stones appeared in the left side of 51 cases (45.5%), and the right side in 61 cases (54.5%). A KUB examination was carried out for all patients before the operation, confirming that they were all positive.

## Operation details

Puncture channels of PCNL were all single channels with 18F. Double J tubes and nephrostomy tubes were retained after surgery. The constant-speed pressure limiting pump was used as the water source during the surgery. The pump speed was 680 ml/min and the pressure limit was 700 mmHg. The model of the holmium laser surgery system used was Auriga XL (StarMedTec GmbH Company) produced on August 19, 2016. During lithotripsy, the core diameter was 600 um, the lithotripsy mode was used, and the frequency was 12 Hz. The energy of a single pulse was 3500 mJ. After lithotripsy, the number of pulses, lithotripsy time (s), and lithotripsy energy (mJ) were read from the memory of the holmium laser machine. The surgery was performed by two doctors independently. The surgery was performed by Wang Yunyan in 69 cases, (61.6%) and by Ji Lu in 43 cases (38.4%).

All patients received CT urography (CTU) examination before surgery. In CTU, the distance of 0.7 mm and the plane thickness of 1 mm were set as references. The stones were measured by two experienced clinicians, each measuring the calculi once. The results were averaged. The *Q* was measured using a CTU non-contrast sequence with the following methods. From top to bottom, the first level of the stone was recorded as 1 and the second level as 2. The 7th (4.9 mm), 14th (9.8 mm), 21st (14.7 mm), and other levels of the whole stole were measured, respectively. Then, the levels were measured again from bottom to top with this method, and the results of both times of measurement were averaged. In the plain CT scan, the levels of each visible stone were directly measured. If there were multiple stones, each of them was measured and calculated, and the results were summed. If a stone has several discontinuous surfaces at the same level, the results on these surfaces should be added; and if the stone disappeared before the 7th level, only the maximum cross-section was recorded. If its maximum cross-sections *x* and *y* edges were less than 5 mm in length, the stone was ignored.

The HU values were measured by selecting the circular area of interest within the largest section of the stone on the CT scan image and reading its average HU value. The HU values were between 375 and 1653 HU, with a mean of 1162.51 ± 176.63 HU. HU values less than or equal to 677.5 are considered as soft stone types, HU values greater than or equal to 970 are considered as hard stone types, and those between 677.5 and 970 are considered as mid-hard stone types [13, 14]. There were 90 cases (80.4%) of hard stones, 13 cases (11.6%) of mid-hard stones and 9 cases (8.0%) of soft stones.

The non-contrast CT images in the CTU examination and images obtained from the ordinary non-contrast CT were imported into Mimics21 software. The region of interest was selected in the bone interval of CT value for 3D reconstruction, and the volume, surface area, and other data were read directly from the reconstructed model.

## Statistical methods

The statistical software SPSS26.0 was used, and the measurement data were expressed as *x* ± s. The difference was statistically significant when *p* < 0.05.

## Results

### Information of stones

The 112 patients received 78 to 26,022 pulses, with a mean of 6342.65 ± 5385.40 pulses. The lithotripsy time was between 6.50 and 2168.50 s, with a mean of 528.55 ± 448.78 s. The volume was between 237.64 and 15,027.84 mm^3^, with a mean of 3634.47 ± 3319.30 mm^3^. The *Q* was 4–519 u, with a mean of 122.66 ± 105.06 u, in the top-down group, and 4–532 u, with a mean of 121.71 ± 105.18 u in the bottom-up group. The *Q* was 4–525.5 u, with a mean of 122.18 ± 104.98 u in the average group.

## Linear regression between stone volume and *x* and *y* values

**Fig. 2:**
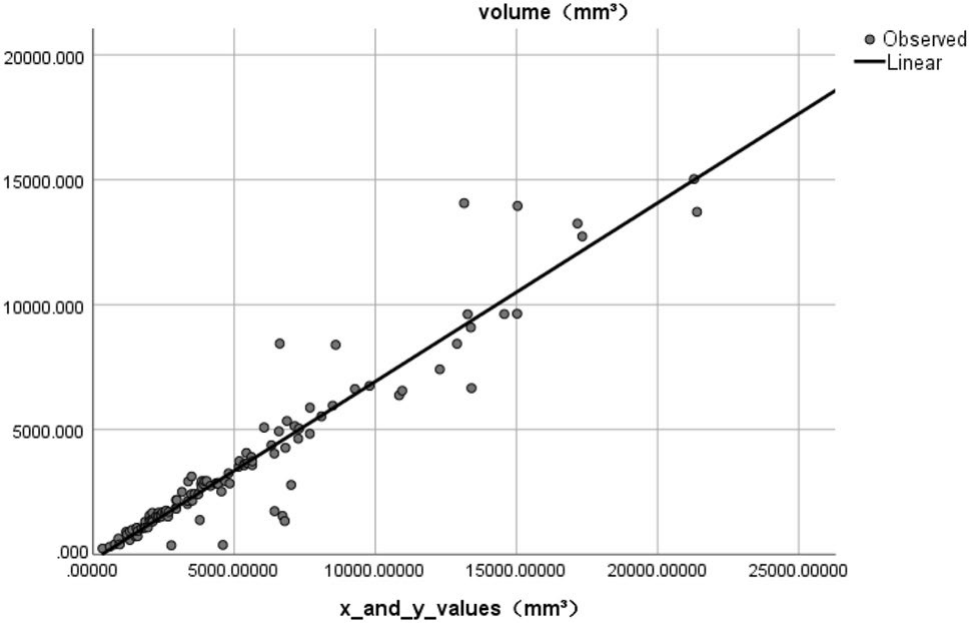
Scatter plots

**Fig. 3:**
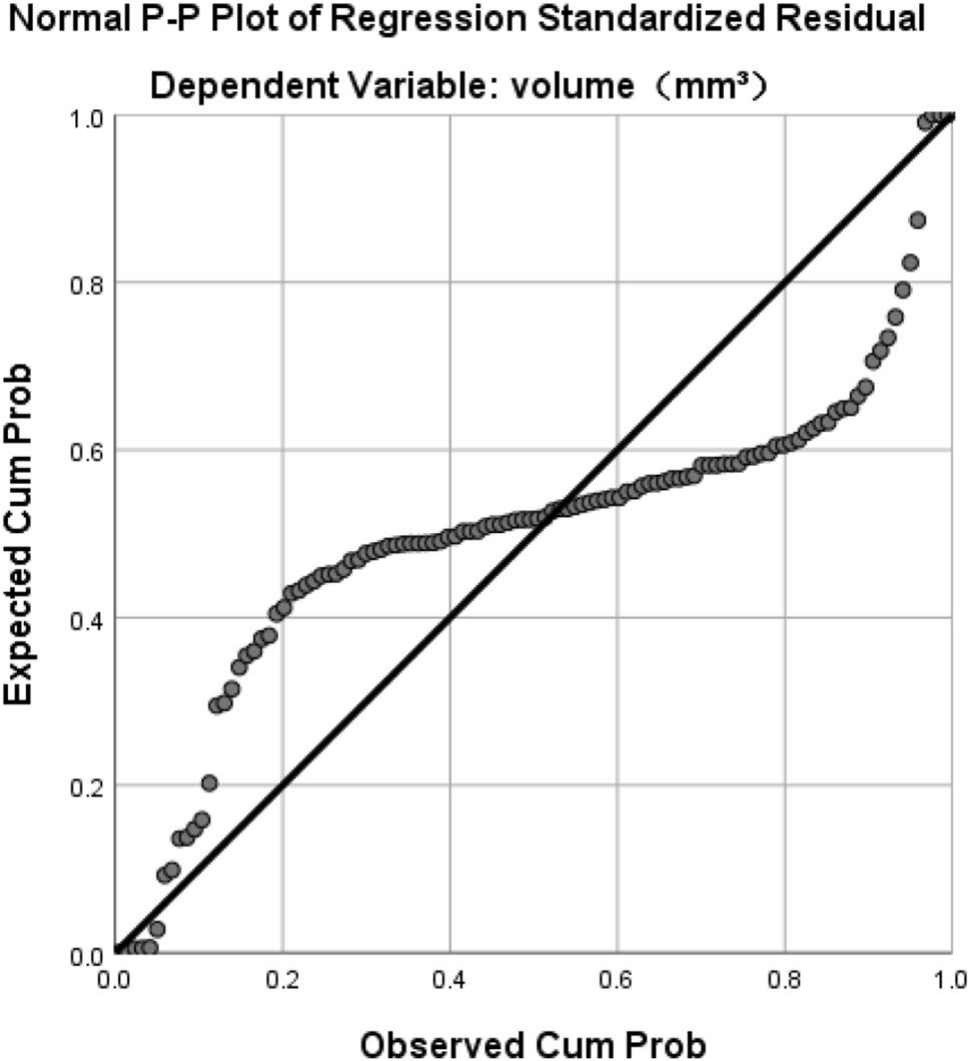
Linear relationships

We multiplied the *x* and *y* values measured by the orthogonal measurement method, and then multiplied both by 0.49 mm. After layer-by-layer accumulation, we calculated the linear regression between both values and stone volume in 3D-reconstructed model: regression coefficient B = 0.715, *R*^2^ = 0.900, *p* < 0.001 (Tables 1, 2, Figs. 2, 3).

**Table 1:** Model summary

Model	*R*	*R*^2^	Adjusted *R*^2^	Std. error of the estimate
1	0.949^a^	0.901	0.900	1051.552191

Dependent variable: volume (mm^3^)

^a^Predictors: (constant), *x*_and_*y*_values (mm^3^)

**Table 2:** Coefficients

Model		Unstandardized Coefficients		Standardized Coefficients	t	Sig
		B	Std. error	Beta		
1	(Constant)	− 222.119	157.499		− 1.410	0.161
	*x*_and_*y*_values (mm^3^)	0.715	0.023	0.949	31.559	0.000

Dependent variable: volume (mm^3^)

The results showed that 90.0% of the stones could be measured using the *x*/*y* layer-by-layer orthogonally. The final volume could be calculated by multiplying the cumulative volume by the regression coefficient *k*. The coefficient of these stones was *k* = 0.715, near to *π*/4, indicating that 90.0% of the stones were ellipse-shaped. This model could be considered as reliable.

## Paired sample *t*-test for *Q* values in top-down and bottom-up groups

The results in showed that there was no statistical difference between the two groups (*p* > 0.05) (Table 3). It was found that the bias from the cross-section selection during the non-contrast CT had not influence on the results of the model.

**Table 3:** Paired sample *t*-test results

Variable name	Top-down group	Bottom-up group	*t*	*p*
Amount of stone (*Q*)	122.66 ± 105.06 u	121.71 ± 105.18 u	0.933	0.353

### Linear relationship between lithotripsy time and *Q*

**Fig. 4:**
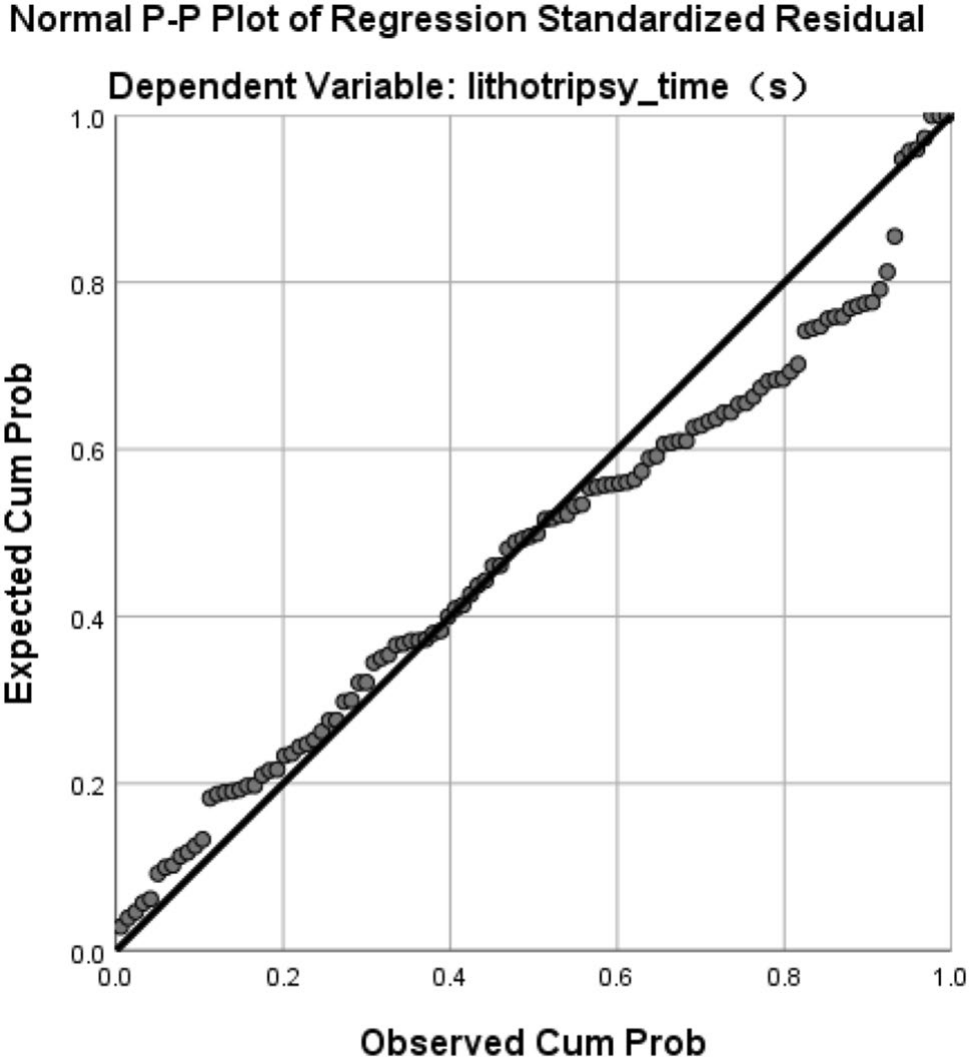
Linear relationships

Excluding age, weight, BMI and other non-relevant data, we believed that the main factors affecting the lithotripsy time are operator and stone type. We set the operator and stone type as dummy variables. The multiple linear regression analysis was performed, using lithotripsy time as a dependent variable, and *Q*-average, operator and stone type as independent variables (Table 4, Fig. 4).

**Table 4:** Multiple linear regression analysis result

Dependent variable: lithotripsy time				
	*Beta*	*t*	*p*	VIF
Amount of stone (*Q*)	0.852	16.438	0.000***	1.051
Operator-wyy	0.110	2.162	0.033*	1.015
Stone type-mid	0.029	− 0.561	0.576	1.027
Stone type-soft	− 0.059	− 1.137	0.258	1.062
Adjusted * R*^2^	0.716			
*F*	70.960***			

*p < 0.05; **p < 0.01; ***p < 0.001

The results showed a linear relationship between lithotripsy time and *Q*-average (*p* < 0.01), and a significant correlation between lithotripsy time and operator (*p* < 0.05), but no significant correlation between lithotripsy time and stone type (*p* > 0.05).

### Linear relationship between lithotripsy time and 3D-reconstructed stone volume

**Fig. 5:**
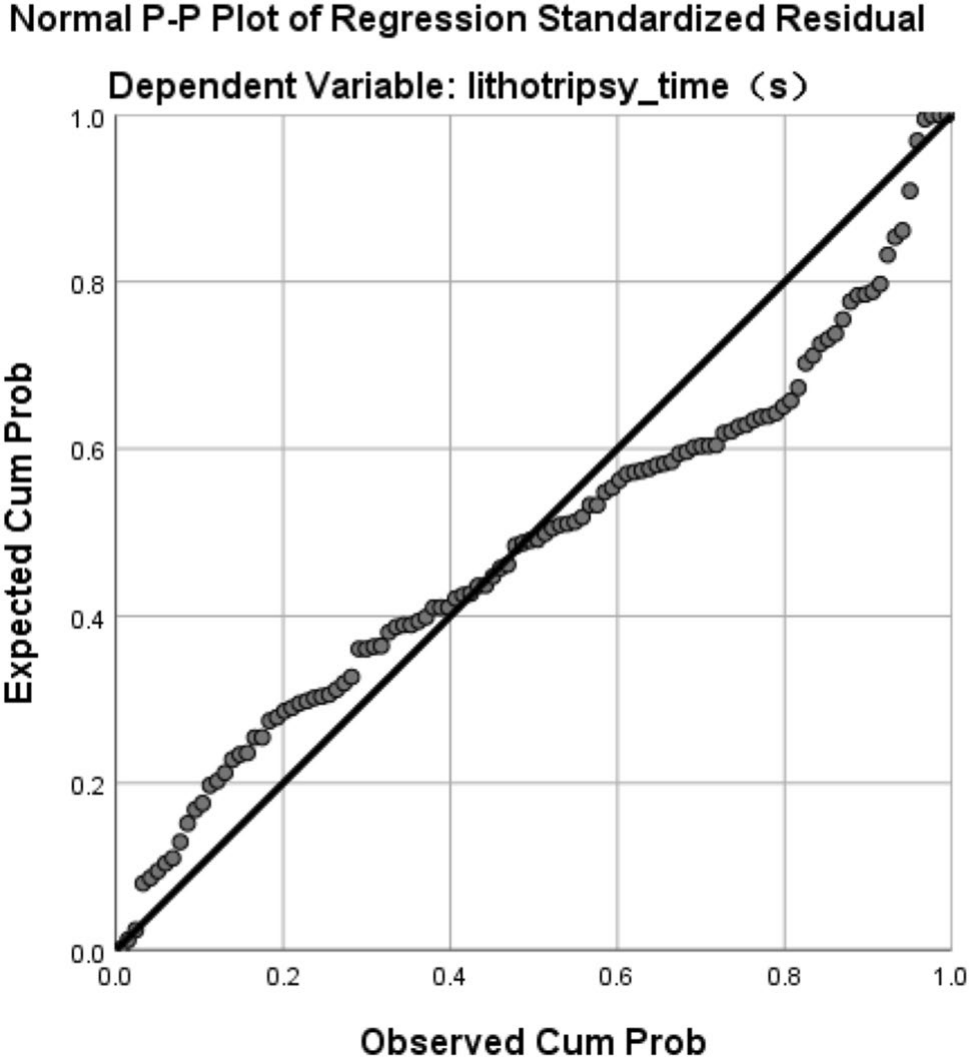
Linear relationships

The regression analysis was repeated with 3D-reconstructed stone volume as an independent variable (Table 5, Fig. 5).

**Table 5:** Multiple linear regression analysis result

Dependent variable: lithotripsy time				
	*Beta*	*t*	*p*	VIF
Volume	0.818	14.467	0.000***	1.048
Operator-wyy	0.175	3.145	0.002**	1.016
Stone type-mid	− 0.022	− 0.396	0.693	1.026
Stone type-soft	− 0.046	− 0.802	0.425	1.058
Adjusted * R*^2^	0.661			
*F*	55.186***			

*p < 0.05; **p < 0.01; ***p < 0.001

The results showed a linear relationship between lithotripsy time and 3D-reconstructed stone volume (*p* < 0.01), and a significant correlation between lithotripsy time and operator (*p* < 0.05), but no significant correlation between lithotripsy time and stone type (*p* > 0.05).

### Linear relationship between lithotripsy time and maximum diameter

**Fig. 6:**
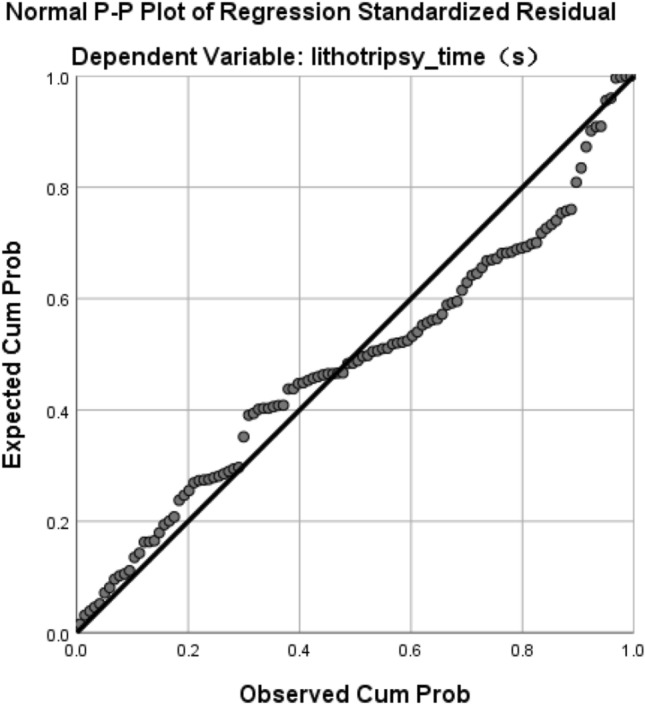
Linear relationships

The regression analysis was repeated with maximum diameter as an independent variable (Table 6, Fig. 6).

**Table 6:** Multiple linear regression analysis result

Dependent variable: lithotripsy time				
	*Beta*	*t*	*p*	VIF
Max diameter	0.692	9.776	0.000***	1.053
Operator-wyy	0.076	1.087	0.279	1.023
Stone type-mid	0.050	0.717	0.475	1.027
Stone type-soft	− 0.012	− 0.165	0.870	1.054
Adjusted *R*^2^	0.471			
*F*	25.722***			

*p < 0.05; **p < 0.01; ***p < 0.001

The results showed a linear relationship between lithotripsy time and maximum diameter (*p* < 0.01), but no significant correlation between lithotripsy time and operator (*p* > 0.05), and no significant correlation between lithotripsy time and stone type (*p* > 0.05).

### Result analysis

The lithotripsy time was significantly related to the independent variables (maximum diameter, stone volume, and *Q*) (*p* < 0.01), suggesting that the regression equations were valid. Therefore, with independent variables unchanged, we can grade the linear fit in the three groups of regression equations by comparing the size of *R*^2^. The fit was the highest in the *Q* group (*R*^2^ = 0.716), moderate in the stone volume group (*R*^2^ = 0.661), and low in the maximum diameter group (*R*^2^ = 0.471). The *Q* was more accurate than stone volume and maximum diameter in estimating the cost of stone-crushing and predicting the lithotripsy time.

Since the operators had a significant effect on the results in the regression analysis for both *Q* and volume groups (*p* < 0.05), we divided the data into two groups according to the operator and redid the regression analysis separately, and came out with the results in Table 7. The *R*^2^ for the two groups were 0.690 (*Q*), 0.648 (volume) and 0.770 (*Q*), 0.745 (volume), respectively. It is consistent with the above results.

**Table 7:** The adjusted *R*^2^ between two groups

	Amount of stone (*Q*)	Volume
Operator-wyy	0.690	0.648
Operator-gl	0.770	0.745

## Discussion

Bleeding and infection are the most common surgical complications of percutaneous nephrolithotomy [15]. A long-time lithotripsy may cause infection, bleeding and other adverse events [16]. The risk of infection increases obviously if the lithotripsy time in percutaneous nephrolithotomy exceeds 90 min [17]. Our study confirmed that amount of stone, measured by the mesh model, is accurate in predicting the lithotripsy time before the operation. Moreover, its accuracy was significantly superior than that of stone volume and maximum diameter measured by the 3D reconstruction.

The time of percutaneous nephrolithotomy includes not only the lithotripsy time, but also the extraction time. The number of targeted stone units directly affected the extraction time, as shown by our mesh model. The eliminate speed of smaller stone fragments through 18F is faster than that of 5 mm, so there maybe an optimal balance point between the size of gravel and sheath. No obvious evidence could confirm the relationship between the number of the targeted units and the extraction time in the present study, which will be revealed by the follow-up study.

The stone volume recommended by the previous scholars has a limited application in clinic, because the measurement requires specific 3D imaging software. The amount of stone in our study can be measured based on the non-contrast CT. Although the model formula looked complicated, the calculation of the results is relatively simple. For example, a stone with a very big size of about 4 × 4 cm can be seen on 8 levels of CT, the (*x*_*n*_, *y*_*n*_) are (22.63,18.08, (19.73,19.80), (17.39,16.81), (18.15,12.36), (30.41,17.15), (35.78,18.08), (36.42,18.21), and (27.21,16.97), it only takes within 3 min to measure it. After unitization with 5 mm as the standard (that is, rounding after 2 times), they are (5, 4), (4, 4), (4, 4), (4, 3), (7, 4), (8, 4), (8, 4), and (6, 4), The calculation is (4 × 4 + 5 × 3) + 4 × 4 + (3 × 4 + 4 × 3) + 4 × 4 + (3 × 4 + 4 × 3) + 4 × 3 + (4 × 2 + 3 × 3) + 4 × 3 + (7 × 3 + 6 × 4) + 7 × 4 + (7 × 4 + 8 × 3) + 8 × 4 + (7 × 4 + 8 × 3) + 6 × 4 + (5 × 4 + 6 × 3) = 423 u, it only takes within 5 min to calculate it and it is more efficient to calculate small stones.

The skill of the operator will have an impact on the efficiency of the lithotripsy. The mesh model’s actual target lithotripsy time is the theoretical minimum lithotripsy time, which the actual operator cannot achieve. The better the operator's operating skills, the closer he can approach this time, and the higher the linear fitting when doing linear regression. In this study, the difference of lithotripsy time between the two operators was statistically significant (*p* < 0.05).

The mesh model can also applied to calculate the amount of stones reduced into target size when the channel of percutaneous nephrolithotomy is less than 18F. Meanwhile, reducing the spacing of preoperative CT scans to the corresponding size is necessary to make the accurate measurement. The lithotripsy time of different channels can be successfully predicted by quantifying different target sizes of stones, which can provide reference for selecting the intraoperative channel size.

The *Q* can be calculated based on the shape of the section. For example, the length and width, instead of diagonal maximum diameter, can be measured directly for the relatively regular section, such as the section close to a rectangle. For the irregular stone sections, special treatment can be implemented. For example, the T-shaped or L-shaped stone sections can be regarded as two connected rectangular stones, which can increase the accuracy of the results.

Many scholars have proposed many other scoring systems to quantify stones, but their usage is restricted due to lack of unified standards and theoretical evidence. For example, the S.T.O.N.E scoring system proposed by Okhunov [18], where the character S represents the maximum cross-sectional area of calculi, was not only difficult to operate but also was inaccurate.

In the process of lithotripsy, the single energy often spills over, which may explain that there was no obvious correlation between the operation time and stone type and single energy in this study.

Due to the small number of cases included in our study, the optimal single energy could not be calculated, which should be resolved by further large-sample studies.

Our study was a single-center study with a small number of cases, which may lead to the selective bias and have a certain impact on the analysis of the results.

The powder lithotripsy and the popcorn technology are often applied in the rigid ureteroscopy and the flexible ureteroscopy [19, 20], which leads to ineffective pulses during the operation, so it is not suitable to use this method for research. We collected some data of ureteroscopic holmium laser lithotripsy, but failed to obtain the effective results.

## Conclusion

The amount of stone (*Q*) was obviously superior to stone volume and maximum diameter in predicting the lithotripsy time, which can become a new index for evaluating stones in the future.

## Data Availability

The data that support the findings of this study are available on request from the corresponding author, upon reasonable request.
